# Re: Detailed association between B‐type natriuretic peptide levels and ventricular tachyarrhythmia

**DOI:** 10.1002/clc.24145

**Published:** 2023-10-23

**Authors:** Nur Sourour, Egil Riveland, Torbjørn Omland, Harald Kjekshus, Alf Inge Larsen, Helge Røsjø, Peder Langeland Myhre

**Affiliations:** ^1^ Department of Cardiology, Division of Medicine Akershus University Hospital Lørenskog Norway; ^2^ K.G. Jebsen Center for Cardiac Biomarkers, Institute of Clinical Medicine University of Oslo Oslo Norway; ^3^ Department of Cardiology Stavanger University Hospital Stavanger Norway; ^4^ Institute of Clinical Sciences University of Bergen Bergen Norway; ^5^ Division for Research and Innovation Akershus University Hospital Lørenskog Norway

To the Editor,

We have read the correspondence to our article by Drs. Kataoka and Imammura with interest and are grateful for the insightful comments related to three aspects of our study, which assessed the association between N‐terminal pro‐B‐type natriuretic peptide (NT‐proBNP) and incident ventricular arrhythmias (VAs).^1^

The first comment relates to subgroups that have been shown to benefit more from implantable cardioverter defibrillator (ICD), that is patients with ischemic cardiomyopathy and patients in New York Heart Association (NYHA) functional class II.^2^ In our Cox multivariable regression models, we adjusted for several a priori selected potential confounders, including coronary artery disease, heart failure (HF) and left ventricular ejection fraction. However, we did not adjust specifically for ischemic cardiomyopathy and NYHA class, but we concur that this is relevant and have performed additional analyses. We find that the association between NT‐proBNP and the risk of incident VA persists after further adjustment for ischemic cardiomyopathy and NYHA class: hazard ratio (HR): 1.33 (95% confidence interval [CI]: 1.04–1.69) per log‐unit increase in NT‐proBNP concentration, *p* = .02. Moreover, the results were consistent in primary prevention ICD patients with ischemic versus nonischemic HF etiology (*p* = .32 for interaction). There was no interaction by the NYHA class on the association (*p* = .24 for interaction). These findings suggest that NT‐proBNP concentrations predict VA risk irrespective of NYHA class and HF etiology.

The second comment relates to the time from ICD implantation to the baseline measurements of NT‐proBNP in our study. We recognize that this may play a role as the beneficial effects of ICD seem to decline over time.^2^ The median time from ICD implantation to the baseline visit in the SMASH 1 Study was 3.7 (Q1‐3 1.4–7.1) years. Additional adjustment for time since ICD implementation did not significantly change the association between NT‐proBNP concentrations and incident VA: HR: 1.22 (95% CI: 1.03–1.45), *p* = .02. However, we did find an interaction (*p* = .039) between time since ICD implantation and NT‐proBNP and risk of VA, such that the association was stronger in patients with a longer time since ICD implant. For patients with ≥3.7 years since implantation the association was highly significant (HR: 1.63 [95% CI: 1.36–1.96], *p* < .001, while it was nonsignificant for patients with <3.7 years since implantation (HR: 1.18 [95% CI: 0.99–1.42], *p* = .07). This may suggest that the importance of NT‐proBNP for VA risk stratification increases with time since ICD implantation.

The third comment relates to our outcome measure VA and its subgroups, which may have different pathophysiological mechanisms. We have data on ventricular tachycardia (VT) versus ventricular fibrillation (VF), but unfortunately not regarding the subtypes of VT (monomorphic vs. polymorphic). In the fully adjusted model, we find that higher concentrations of NT‐proBNP were significantly associated with *both* the risk of VT (HR: 1.39 [95% CI: 1.21–1.59], *p* < .001) and the risk of VF (HR: 1.50 [95% CI: 1.20–1.88], *p* < .001). Furthermore, NT‐proBNP concentrations were significantly associated with both events that required anti‐tachycardia pacing (HR: 1.41 [95% CI: 1.22–1.63], *p* < .001) and events that required electrical defibrillation (HR: 1.43 [95% CI: 1.18–1.73], *p* < .001). We believe these additional analyses support NT‐proBNP measurement as a robust predictor of incident VA, especially in patients with a secondary prevention ICD indication.^1^
